# Natural Volatile Compounds as Antifungal Agents Against *Monilinia fructicola* In Vitro and in Composite Edible Coatings for Sustainable Disease Reduction and Fruit Quality Preservation During Prolonged Cold Storage of Fresh Japanese Plums

**DOI:** 10.3390/foods14234088

**Published:** 2025-11-28

**Authors:** María Victoria Alvarez, Lluís Palou, Verònica Taberner, María Bernardita Pérez-Gago

**Affiliations:** 1 Grupo Investigación en Ingeniería en Alimentos, Instituto de Ciencia y Tecnología de Alimentos y Ambiente (INCITAA), Facultad de Ingeniería, Universidad Nacional de Mar del Plata, Consejo Nacional de Investigaciones Científicas y Técnicas (CONICET), Mar del Plata 7600, Argentina; mvalvarez@fi.mdp.edu.ar; 2 Centre de Tecnologia Postcollita (CTP), Institut Valencià d’Investigacions Agràries (IVIA), 46113 Montcada, València, Spain; taberner_ver@gva.es (V.T.); perez_mbe@gva.es (M.B.P.-G.)

**Keywords:** *Prunus salicina*, postharvest, brown rot, antifungal plant extracts, essential oils, bio-based edible coatings, plum quality

## Abstract

The antifungal activity of natural extracts, essential oils (EOs), and pure volatiles against *Monilinia fructicola*, the main causal agent of brown rot of stone fruits, was evaluated in in vitro tests. Cinnamon (CI), lemongrass (LE), geraniol (GE), and myrrh (MY) EOs were the most effective antifungal agents and, hence, selected as ingredients of composite edible coatings (CECs) formulated with hydroxypropyl methylcellulose (HPMC) and lipidic components. In in vivo curative experiments with ‘Black Gold’ plums artificially inoculated with *M. fructicola* and incubated for up to 10 days at 20 °C, brown rot incidence was reduced by up to 49% with CECs containing 4 g/kg LE, 2 g/kg GE, or 5 g/kg MY. These CECs were then evaluated for brown rot control and quality maintenance of ‘Angeleno’ plums stored for up to 6 weeks at 1 °C and 90% RH, followed by a shelf-life period of 4 days at 20 °C. After 3 weeks, CECs containing GE and MY reduced brown rot incidence by 45 and 70%, respectively. After 6 weeks plus shelf life, all CECs reduced brown rot severity (lesion size) by 30–50%. Regarding fruit quality, coated plums showed higher firmness than uncoated control plums, and the CEC containing GE significantly reduced weight loss after 6 weeks plus shelf life. Moreover, physicochemical quality attributes (titratable acidity, soluble solids content, and volatile compounds) and sensory properties (overall flavor, off-flavor, firmness, and external appearance) of coated plums were not negatively affected by CEC application. Furthermore, all coated plums exhibited more gloss than uncoated fruit. Overall, the CEC-GE formulation was the most effective in reducing decay and maintaining the postharvest quality of cold-stored plums, showing the best potential as a sustainable alternative for plum postharvest preservation.

## 1. Introduction

Japanese plums (*Prunus salicina*) are a summer fruit crop widely cultivated worldwide and highly appreciated by consumers due to their sensory quality and valuable nutritional properties, containing vitamins, minerals, and antioxidants such as anthocyanins and flavonoids, among other nutrients [1]. However, their storage and shelf life are considerably limited by high susceptibility to chilling injury, firmness loss, and postharvest diseases caused by pathogenic fungi. In general, the optimal temperature for cold storage of plums is 0–1 °C. However, excessive storage at this temperature, as well as exposure to temperatures between 2 and 7 °C, often reached during fruit transportation, can result in chilling injury. Symptoms of this damage are evidenced in the plum flesh as a loss of juiciness, vitrification, internal browning, and/or the appearance of red stains or ‘bleeding’ due to the release of anthocyanins. The development of these symptoms typically occurs when fruits are stored at room temperature after the cold storage period, meaning that the disorder is generally detected directly by consumers, with the consequent impact on their subsequent purchasing decisions [2].

Brown rot (BR), caused by *Monilinia* spp., is the most economically significant disease affecting stone fruits during the postharvest period [3]. Infections by *Monilinia* spp. are initiated in the field and may remain latent until harvest. The incidence of BR can be successfully controlled by employing chemical treatments with effective fungicides before and after harvest [4]. However, current strategies for managing fruit diseases demand a significant reduction in the reliance on conventional fungicides. This trend is primarily driven by increasing concerns about environmental pollution and potential human health risks associated with chemical residues in fruits [3]. Furthermore, the emergence of fungicide-resistant pathogenic strains has diminished the effectiveness of several synthetic compounds, emphasizing the urgency to develop alternative approaches for disease control [4]. Therefore, there is great interest in finding natural and sustainable alternatives to control postharvest BR of stone fruits, while preserving fruit quality. In this regard, different postharvest strategies have been tested as alternatives to synthetic fungicides, including physical treatments, biological control, and low toxicity chemical treatments such as the application of food additives, essential oils (EOs), natural extracts, and edible coatings (ECs) formulated with nonpolluting antifungal agents.

Although several of these technologies have proven effective in different studies, further research is still needed to advance their commercial implementation [3]. Thus, for example, several works have reported that thyme, oregano, and savory EOs [5], cinnamon and clove EOs [6], rose EO [7], and pomegranate peel extracts [8], applied as aqueous solutions, controlled BR to varying degrees on different stone fruits such as apricots, nectarines, and plums. Also, in other studies, in vivo BR development was reduced by applying vapor treatments of lemon myrtle [9], fennel, basil, and lemon EOs on nectarines [10] and thyme and savory EOs on peaches and nectarines [11]. However, significant limitations of these applications relate mainly to adverse effects on the sensory quality of treated fruits; phytotoxicity, which depends on the volatile concentration and the sensitivity of each species and cultivar of stone fruits, and reduced effectiveness of these agents when applied in vivo compared to that observed in in vitro tests [6,9,11,12]. Notably, the development of ECs has gained considerable attention in recent years as an environmentally friendly and safe technology for prolonging the shelf life of fresh and minimally processed fruits. These coatings act as semi-permeable barriers to gases and water vapor, contributing to a reduction in the fruit’s metabolic activity, maintaining firmness, and reducing weight loss, among other benefits [13]. In general, these functionalities of ECs are provided by different ingredients of the coating matrix, a hydrocolloid to regulate gas exchange and a lipid to regulate water vapor exchange, and these complex ECs are known as composite edible coatings (CECs). In addition, CECs can be further functionalized by incorporating active compounds including antimicrobial agents. When enriched with natural alternative antifungals such as food additives, natural extracts, and EOs, the resulting CECs can contribute to the effective control of postharvest diseases [14]. As an additional advantage, the use of CECs as carriers of natural antifungal agents may offer solutions to the limitations of the direct use of these substances on fresh fruits mentioned above [12]. Therefore, the postharvest application of CECs to fresh plums can provide a dual effect in a single postharvest treatment, regulating fruit physiology during storage and controlling fungal decay, which could contribute to a significant reduction in the economic losses for fruit producers and retailers.

Several studies reported the efficacy of CECs enriched with natural antifungal compounds, including EOs and natural extracts, to improve postharvest quality of plums [14,15,16,17,18,19,20,21]. However, research focused on the development of this type of coatings based on the control of brown rot caused by *Monilinia* spp. as the primary functionality is scarce. Recently, a gum Arabic-based CEC enriched with thymol and salicylic acid was tested to reduce BR caused by *Monilinia laxa* (Aderh. & Ruhland) Honey, on ‘Angeleno’ plums [21]. Similarly, Asgarian et al. [14] reported significant reductions in the incidence and severity of BR caused by *Monilinia fructicola* (G. Winter) Honey during cold storage of ‘Angeleno’ plums by the application of hydroxypropyl methylcellulose (HPMC) and gum Arabic-based coatings containing geraniol. A large number of EOs and natural extracts from plant or animal origin remain unexplored regarding their inhibitory capacity against *M. fructicola* and their performance as antifungal ingredients for the functionalization of biobased coatings. Such antifungal CECs could replace the use of synthetic commercial waxes containing chemical fungicides after harvest of stone fruits. In the present research, a group of natural substances such as EOs, pure volatile compounds, and natural plant extracts were studied as potential ingredients for the development of antifungal CECs based on HPMC and glyceryl monostearate (GMS) to control BR and maintain the postharvest quality of plums. The objectives of this work were to (i) investigate the in vitro activity of natural substances against *M. fructicola* and select the most effective ones; (ii) assess the curative activity of CECs formulated with selected natural antifungals against BR on artificially inoculated plums incubated at 20 °C; (iii) study the impact of selected antifungal CECs on BR development on inoculated and long-term cold-stored plums; and (iv) evaluate the impact of these treatments on the physicochemical and sensory quality of plums stored for 3 and 6 weeks at 1 °C, followed by 4 days at 20 °C, simulating commercial conditions of fruit cold storage and shelf life.

## 2. Materials and Methods

### 2.1. Antifungal Agents and Coating Materials

Geraniol (GE), eugenol (EU), the EOs from cinnamon (CI, *Cinnamomum zeylanicum*) and lemongrass (LE, *Cymbopogon citratus*), and vanillin (VA) were purchased from Sigma-Aldrich (St. Louis, MO, USA). Myrrh (MY, *Commiphora myrrha*) EO was provided by Essenciales (Barcelona, Catalonia, Spain) and savory (SA, *Satureja montana*) EO by Essential’ Arôms (Lleida, Catalonia, Spain). Green tea (GT) dry extract was purchased from Guinama (Valencia, Spain). Hydroxypropyl methylcellulose (HPMC; Methocel E19, Dow Chemical Co., Midland, MI, USA) was used as biopolymer in the coating formulations while the lipidic phase was composed of glyceryl monostearate (GMS, Monestriol, Italmatch Chemicals Spa, Barcelona, Catalonia, Spain). The emulsifiers sunflower lecithin (Giralec HE60) and diacetyl tartaric acid esters of mono-diglycerides (Moglicet G) were provided by Lasenor (Barcelona, Catalonia, Spain). Glycerol was purchased from Panreac Química (Barcelona, Catalonia, Spain).

### 2.2. Preparation of Monilinia fructicola Inoculum

In this study, the strain MeCV-2 of the fungal pathogen *Monilinia fructicola* (G. Winter) Honey was used. This strain, isolated from a decayed peach fruit found in a packinghouse located in the Valencia region (Spain), is available at the IVIA culture collection of postharvest pathogens and it is also deposited, with the accession number CECT 21161, in the Spanish Type Culture Collection (CECT, University of Valencia, Valencia, Spain). After growing on Petri dishes containing potato dextrose agar (PDA) (Scharlab S.L., Barcelona, Spain) for 7–14 days at 25 °C, spore suspensions at high concentrations were prepared using the methodology described by Karaca et al. [13] to be used in both in vitro and in vivo assays.

### 2.3. In Vitro Antifungal Activity of Natural Compounds

In vitro mycelial growth inhibition was evaluated in PDA Petri dishes using two different methods depending on the nature of the tested antifungal agents [22]. For the evaluation of volatile compounds (GE, EU, CI, LE, and SA) in the vapor phase, doses of 5 and 10 µL of volatile were used to soak 55 mm diameter sterile filter paper disks that were placed in the lid of 90 mm diameter PDA Petri dishes inoculated with *M. fructicola*. After sealing with Parafilm, dishes were incubated upside-down. Sterile distilled water was used as control treatment. Non-volatile natural extracts (GT and VA) and MY EO were evaluated by direct contact using the agar dilution method. Pure dimethyl sulfoxide (DMSO) was used to dissolve VA and MY before the assay while GT was dissolved in sterile distilled water. Appropriate amounts of these solutions were added to the culture medium to achieve the following concentrations: 5, 10, and 20 g/kg for GT; 0.31, 0.62, and 1.25 g/kg for VA; and 0.62, 1.25, and 2.5 g/kg for MY. Petri plates with PDA alone and PDA containing DMSO (2.5 g/kg) were prepared as control treatments. In all cases, the inoculation of each Petri dish was performed by adding 20 µL of a spore suspension of *M. fructicola* (10^4^ spores/mL) in the center of the agar. Inoculated plates were incubated for 7 days at 25 °C in the dark. Radial mycelial growth was determined in each plate by averaging two perpendicular fungal colony diameter measurements. Five replicates were used for each antifungal agent and concentration. Results were expressed as the percentage of mycelial growth inhibition related to the fungal growth in the corresponding control plates (i.e., PDA alone for GT plates and PDA + DMSO for VA and MY plates) [23]. For those Petri dishes showing 100% fungal growth inhibition, in order to figure out if the effect was transient or permanent (lethal), the effective fungicidal effect of the agent was evaluated as reported by Alvarez et al. [23] for volatiles and by Sellamuthu et al. [24] for non-volatile antifungals tested by direct contact.

### 2.4. Preparation of Composite Edible Coatings (CECs) with Antifungal Properties

CECs were prepared as emulsions using HPMC as hydrophilic phase and GMS as lipidic phase. The base emulsion was formulated by combining a HPMC solution (34 g/kg) with GMS (7.7 g/kg), Giralec HE-60 (7.7 g/kg), and Moglicet (7.7 g/kg). Glycerol (5 g/kg) was added as a plasticizer. The mixture was heated to 92 °C and homogenized using a high-shear probe mixer (Ultra-Turrax IKA^®^ model T25, IKA-Werke, Staufen, Germany) for 1 min at 12,000 rpm plus 3 min at 22,000 rpm. Then, the emulsions were allowed to cool to 20–22 °C before adding the antifungal ingredients. The selection of pure volatiles and EOs for this study was based on the results of the in vitro screening test. First, preliminary in vivo trials were conducted with the preselected antifungal agents as ingredients in different CECs formulations, evaluating important emulsion properties such as stability and viscosity. As a result of this preliminary trial, four effective antifungal agents were selected at two different concentrations, resulting in the following treatments that were tested in vivo (Section 2.6): CEC-GE (1 g/kg), CEC-GE (2 g/kg), CEC-CI (2 g/kg), CEC-CI (4 g/kg), CEC-LE (2 g/kg), CEC-LE (4 g/kg), CEC-MY (5 g/kg), CEC-MY (10 g/kg), and CEC without addition of any antifungal agent (CEC). To prepare the final antifungal CECs, each base emulsion was enriched with the corresponding antifungal ingredient, and these mixtures were homogenized for 1 min at 16,000 rpm. These antifungal CECs resulted in stable emulsions in all cases, with viscosity values ranging from 253 to 372 MPa.s and pH values ranging 2.70–2.73.

### 2.5. Plum Fruit

Plums (*Prunus salicina* Lindl.) cvs. ‘Black Gold’ and ‘Angeleno’ were used for the in vivo assays at 20 °C and at 1 °C, respectively. Plums were harvested in commercial orchards in the Valencia area (Spain), transported to the IVIA, and stored for up to 5 days at 1 °C and 90% relative humidity (RH) before use. Fruits were selected for uniformity of color and size and absence of external damage. Previous to each experiment, fruit were disinfected by immersion in a 0.5% sodium hypochlorite solution for 4 min, rinsed with tap water, allowed to air-dry at room temperature, and randomized before treatment application.

### 2.6. In Vivo Effect of Antifungal CECs on Brown Rot Development

The curative activity of CECs was assessed following the methodology previously described by Karaca et al. [13] and Gunaydin et al. [25] with some modifications. ‘Black Gold’ plums were artificially inoculated with *M. fructicola* (inoculum concentration of 10^3^ spores/mL) using a stainless-steel rod with a probe tip 1 mm wide and 2 mm in length previously dipped into the spore suspension to wound the peel of each fruit once on the equator. After storage for 24 h at 20 °C and 90% RH, a volume of coating formulation (0.4 mL) of the corresponding CEC described in Section 2.4 was deposited onto the peel of each inoculated plum and rubbed with gloved hands to simulate rotary industrial coating application in stone fruit packing lines. Inoculated but uncoated plums served as control treatment. After air-drying at room temperature, treated fruit were incubated in a controlled storage room at 20 °C and 90% RH for up to 10 days. After 3 and 8 days of incubation, disease parameters were evaluated on coated fruit in comparison with control fruit. Brown rot incidence was measured as the percentage of infected wounds and brown rot severity as lesion size (mm diameter). Four replicates of 10 fruit each were used for each treatment.

### 2.7. Impact of Selected CECs on Brown Rot and Quality of Cold-Stored Plums

Based on the results obtained in the previous in vivo experiments, CEC-GE (2 g/kg), CEC-LE (4 g/kg), and CEC-MY (5 g/kg) coatings were selected to further evaluate their ability to control BR and preserve postharvest quality of ‘Angeleno’ plums during cold storage. An uncoated control and the coating matrix without antifungal agents (CEC) were also included. Plums were prepared as described above and randomly divided into two lots: one for the pathology assay for inoculation with *M. fructicola* and the other for evaluation of quality attributes in non-inoculated fruit.

#### 2.7.1. Brown Rot Control on Coated Plums During Cold Storage

The curative activity of CECs on artificially inoculated, coated 24 h later, and cold-stored plums was assessed using the same methodology described in Section 2.6. Coated and uncoated control fruit were stored at 1 °C for 6 weeks, followed by 4 additional days at 20 °C (shelf-life period). BR incidence (percentage) and severity (mm) were evaluated on the same fruits weekly for up to 6 weeks and also at the end of the shelf-life period at 20 °C. Four replicates of 20 fruit each were used for each treatment.

#### 2.7.2. Quality of Coated Plums During Cold Storage

CECs were manually applied to 150 plums per treatment as previously described. For control samples, fruit was dipped in 20 °C tap water for 15 s. At harvest and after 3 and 6 weeks of cold storage at 1 °C and 90% RH followed by a 4-day shelf-life period at 20 °C, the following plum quality attributes were evaluated:

Weight Loss. Weight loss was determined by individually weighing 30 marked plums per treatment at the beginning and at the end of each storage period with a calibrated precision balance (PB3002-S, Mettler Toledo, Cornellà del Llobregat, Catalonia, Spain). Results were expressed as the percentage of initial weight loss.

Flesh Firmness. Plum flesh firmness was measured using an Instron Universal Testing Machine (Model 4301, Instron Limited, Bucks, UK). A penetration test was performed using an 8 mm diameter plunger. The maximum force (N) required to penetrate the fruit flesh was determined after removing a thin disk of skin of approximately 2 cm diameter [25]. For each treatment, 20 plums were used, and the average value was calculated.

External Color. The surface color of plums was recorded with a colorimeter (Model CR-400, Minolta, Tokyo, Japan) using the CIELAB color space. Changes in L*, a*, b*, Chroma (C*), and hue angle (*h*°) were evaluated in 20 plums per treatment with measurements taken at three different locations of each fruit [25].

Internal Quality. Titratable acidity (TA, g/L malic acid) and soluble solids content (SSC, °Brix) were analyzed in three juice samples per treatment, obtained from 10 fruit each [25]. TA was determined by titrating 5 mL of juice with 0.1 M sodium hydroxide to an end point of pH 8.1 using an automatic titrator (Titrator T50, Mettler Toledo, Cornellà del Llobregat, Catalonia, Spain) and SSC with a digital refractometer (model ATC-1, Atago^®^ Co., Ltd., Tokyo, Japan). Maturity index (MI) was calculated as the ratio SSC/TA.

Fermentation Volatiles Content. Acetaldehyde and ethanol contents (mg/L) were analyzed from the headspace of 5 mL of plum juice samples by gas chromatography (Trace Valve Oven, Thermo Fisher Scientific, Inc., Waltham, MA, USA) according to Gunaydin et al. [25]. For each treatment, 3 replicates of juice from 10 plums each were analyzed.

Physiological Disorders. A visual inspection for symptoms of plum chilling injury (fruit flesh browning and/or bleeding) was conducted on fruit halves at the end of each shelf-life period at 20 °C. Browning was graded using a scale from 1 (none, absence) to 5 (extreme severity) and bleeding with a scale from 1 (none, absence) to 3 (severe) [25]. Three replicates per treatment of 10 fruit each were evaluated and results were expressed as mean average indexes.

Sensory Evaluation. Ten semi-trained panelists evaluated the sensory quality of coated and uncoated control plums following the methodology described by Alvarez et al. [23]. The samples were prepared from a minimum of 8 plums per treatment, previously peeled and cut into wedges, randomly numbered with 3-digit codes, and served to the judges at room temperature on plastic trays. Overall flavor was rated using a structured scale from 1 (very poor) to 9 (optimal), the presence of off-flavors was scored from 1 (absence) to 5 (very pronounced), and eating firmness from 1 (very soft) to 5 (very firm). Panelists also evaluated external the appearance of whole plums with a 1–3 scale in which 1 = bad, 2 = acceptable and 3 = good, and visually ranked treated plums from the highest to the lowest gloss.

### 2.8. Statistical Analysis

Data from both in vitro and in vivo experiments were subjected to one-way ANOVA. For disease incidence data, the analysis of variance (ANOVA) was applied to the arcsine of the square root of the percentage of infected fruit in order to assure the homogeneity of variances. In all cases, non-transformed means are shown. Fisher’s Protected Least Significant Difference test (LSD, *p* < 0.05) was conducted to identify significant differences between means. Gloss rank was analyzed using the Friedman test (*p* < 0.05). All statistical analyses were performed with the software Statgraphics Centurion XVII (Statgraphics Technologies Inc., The Plains, VA, USA).

## 3. Results

### 3.1. In Vitro Antifungal Activity of Natural Compounds Against M. fructicola

Inhibition of the mycelial growth of *M. fructicola* exerted by pure volatiles and EOs (volatile exposure tests) and natural extracts (agar dilution tests) after a 7-day incubation period at 25 °C is shown in Table 1. GE and EU pure compounds and CI, LE, and SA EOs were highly and equally effective when applied as volatiles against *M. fructicola*, with 100% radial growth inhibition and fungicidal effect at a dose of 5 µL. MY EO showed moderate activity when tested in the vapor phase, so it was further evaluated using the agar dilution method. Among the natural antifungal agents tested by the agar dilution method, VA was the most effective, inducing 100% growth inhibition and fungicidal effect when applied at 0.31 g/kg, followed by MY, which inhibited fungal growth by 94% at 0.62 g/kg and showed fungicidal action at 2.5 g/kg. In contrast, high concentrations of GT (20 g/kg) were required to obtain a moderate inhibitory activity.

### 3.2. In Vivo Effect of Antifungal CECs on Brown Rot Development

**Figure 1:**
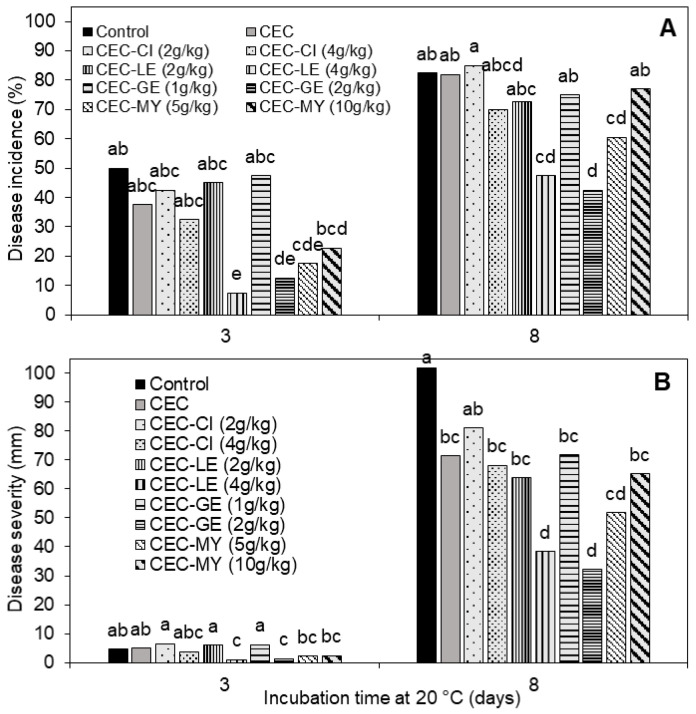
Brown rot incidence (**A**) and severity (**B**) on ‘Black Gold’ plums artificially inoculated with *Monilinia fructicola*, uncoated (Control) or treated 24 h later with composite edible coatings (CECs) containing different concentrations of cinnamon (CI), lemongrass (LE), myrrh (MY) essential oils or geraniol (GE), and stored for 3 and 8 days at 20 °C. Bars indicate mean values (n = 4). For each incubation time, different letters indicate significant differences among treatments according to Fisher’s Protected LSD test (*p* < 0.05) applied after an ANOVA.

In accordance with the results obtained in the previous in vitro tests, the antifungal agents GE, CI, LE, and MY EOs were selected for antifungal CEC formulation and testing in the subsequent in vivo assays. Figure 1 shows the development of BR after 3 and 8 days of incubation at 20 °C on ‘Black Gold’ plums artificially inoculated with *M. fructicola* and coated 24 h later. BR incidence on both uncoated control and CEC-coated (without antifungal agent) plums was high (82–83%) and reached its maximum values after 8 days of incubation (Figure 1A). This fact indicated that the base coating formulation containing HPMC and GMS did not provide antifungal activity by itself, requiring functionalization by the addition of natural antifungal agents. Over the incubation period, CECs containing LE (4 g/kg), GE (2 g/kg), or MY (5 g/kg) significantly reduced disease incidence (*p* < 0.05) compared to control and CEC (without antifungal agent) coatings, with reductions in the ranges of 55–85% and 27–49% after 3 and 8 days at 20 °C, respectively. In contrast, CECs formulated with CI showed no significant reduction in disease incidence compared to the controls (Figure 1A). Regarding disease severity, all coatings significantly reduced BR lesion size throughout the incubation period, and CEC-LE (4 g/kg), CEC-GE (2 g/kg), and CEC-MY (5 g/kg) were the most effective, with severity reductions compared to the uncoated control ranging from 49 to 68% after 8 days (Figure 1B). Therefore, these coatings were selected to be tested under commercial cold storage conditions.

### 3.3. Effect of CECs on Brown Rot and Quality of Cold-Stored Plums

#### 3.3.1. Impact on Brown Rot Development

**Figure 2:**
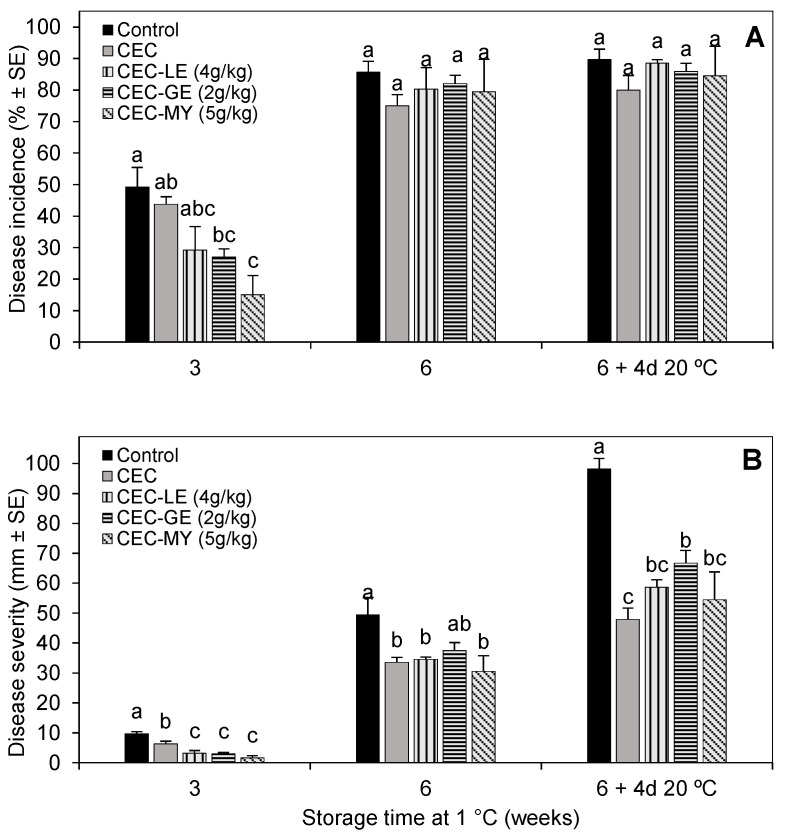
Brown rot incidence (**A**) and severity (**B**) on ‘Angeleno’ plums artificially inoculated with *Monilinia fructicola*, uncoated (Control) or treated 24 h later with composite edible coatings (CECs) functionalized with lemongrass essential oil (LE), geraniol (GE) or myrrh essential oil (MY), and stored for up to 6 weeks at 1 °C and 90% RH followed by a 4-day shelf-life period at 20 °C. Bars indicate mean value ± standard error (SE) (n = 4). For each storage time, different letters indicate significant differences among treatments according to Fisher’s Protected LSD test (*p* < 0.05) applied after an ANOVA.

The development of BR on ‘Angeleno’ plums artificially inoculated with *M. fructicola*, treated with antifungal CECs 24 h later, and stored at 1 °C for up to 6 weeks is shown in Figure 2. After 3 weeks of storage at 1 °C, CEC-GE and CEC-MY significantly reduced BR incidence (*p* < 0.05) by 45 and 70%, respectively, compared to control fruit. However, after 6 weeks of cold storage and 6 weeks plus 4 days of shelf life at 20 °C, BR incidence on uncoated plums reached about 90%, and no significant differences between control and coated fruit were observed (Figure 2A).

All antifungal CECs significantly reduced BR severity (lesion diameter) during the entire cold storage period and shelf life (*p* < 0.05), with severity reductions with respect to the uncoated control in the ranges of 67–83%, 30–38%, and 35–50% after 3 and 6 weeks at 1 °C and after 6 weeks plus shelf life at 20 °C, respectively (Figure 2B). Among the antifungal CECs, CEC-MY-treated (5 g/kg) plums showed the lowest average lesion values during the first 5 weeks of cold storage, but not later. During cold storage, BR severity was equally affected by the antifungal CECs than by the CEC alone (formulated without antifungal agent).

#### 3.3.2. Impact on Postharvest Quality Parameters

##### Weight Loss, Flesh Firmness, and Peel Color of Treated Plums

**Figure 3:**
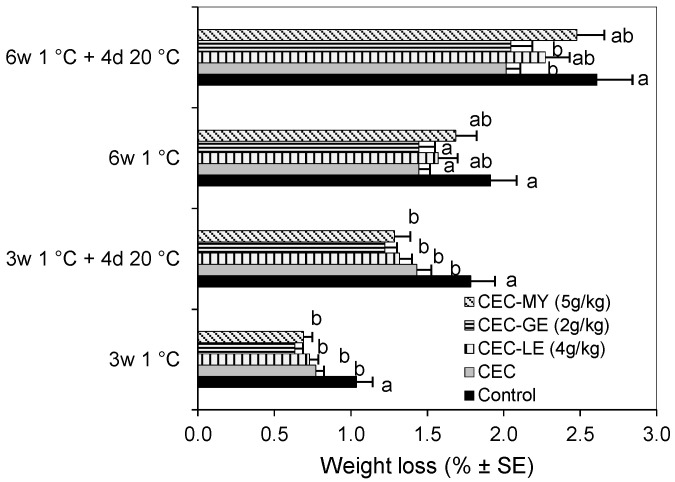
Percentage weight loss of ‘Angeleno’ plums uncoated (Control) or treated with composite edible coatings (CEC) enriched with lemongrass essential oil (LE), geraniol (GE) or myrrh essential oil (MY) and stored for 3 and 6 weeks at 1 °C and 90% RH followed by a 4-day shelf-life period at 20 °C. Bars indicate mean value ± standard error (SE) (n = 30). For each storage time, different letters indicate significant differences among treatments according to Fisher’s Protected LSD test (*p* < 0.05) applied after an ANOVA.

Figure 3 shows the effect of different CECs on plum weight loss during cold storage. As expected, weight loss increased with storage time, reaching a maximum value of 2.61% in uncoated samples (control) stored for 6 weeks at 1 °C plus 4 days at 20 °C. CECs with or without antifungal agents significantly reduced weight loss compared to control fruit after 3 weeks at 1 °C (*p* < 0.05) and after the subsequent shelf-life period. Furthermore, the effect CEC and CEC-GE (2 g/kg) reducing weight loss was maintained until the end of the trial, with reductions of up to 23% compared to the control (*p* < 0.05).

Flesh firmness of ‘Angeleno’ plums at harvest was about 17 N and decreased to values of 12 N on uncoated control fruit and 13–17 N on coated plums at the end of the 6-week cold storage period plus shelf life (Table 2). Although no significant differences were observed between the firmness of uncoated and coated plums after 3 weeks at 1 °C plus 4 days at 20 °C, CEC alone and CECs containing LE and MY maintained firmness significantly higher (*p* < 0.05) than that of the uncoated control after 6 weeks plus shelf life, demonstrating a protective effect of these coatings against fruit softening.

The impact of antifungal CECs on ‘Angeleno’ plum peel color was evaluated after cold storage and shelf life by analyzing the parameters L*, a*, b*, C*, and *h*° (Table 2). As a general trend, a*, b*, C*, and *h*° slightly decreased in both uncoated and coated fruit with storage time, indicating that peel color became less vivid (lower C*) and redder (lower *h*°). In contrast, for each treatment, L* values were maintained throughout storage time. When comparing treatments, coated plums had lower L* values and higher a* and b* values than uncoated plums throughout storage (*p* < 0.05), regardless of the coating formulation applied. Furthermore, all coated samples exhibited *h*° values in the range 3.4–7.9 ° (redder), whereas the control plums showed values between 327 and 351 ° (more purple). On the other hand, no significant differences in L*, a*, b*, C*, and *h*° were found between coating treatments, indicating that the different formulations (with or without essential oils) had the same impact on plum peel color.

##### Internal Physicochemical Quality and Volatile Content

Physicochemical quality attributes of coated and uncoated ‘Angeleno’ plums are listed in Table 3. Plums coated with CECs containing GE and MY showed slightly lower TA values (*p* < 0.05) (0.73–0.75 g/L) compared to uncoated samples (0.84 g/L) after 3 weeks of cold storage plus 4 days at 20 °C, which resulted in higher MI values compared to the control. However, no significant differences between treatments were observed for TA and MI values at the end of the storage period. Similarly, SSC was not significantly affected by CECs application during storage, with values ranging from 12.7 to 14.7 °Brix.

Ethanol and acetaldehyde concentrations in the juice of control plums remained at similar levels over the cold storage period at 1 °C plus shelf life at 20 °C (Table 3). The application of the CEC coating without antifungal agents did not alter the plum volatiles content compared to the uncoated control. In the case of plums coated with EO-enriched CECs, ethanol concentrations were significantly higher (131–312 mg/L) than those found in uncoated and CEC-coated (without antifungal agents) plums (7 and 37 mg/L, respectively) after 6 weeks of cold storage plus shelf life. Regarding acetaldehyde content, no significant differences between treatments were observed after 3 weeks plus shelf life, while higher values (*p* < 0.05) were found in CEC-LE coated plums (7.7 mg/L) compared to control and CEC-coated plums (2.3 and 4.4 mg/L) at the end of the storage (6 weeks plus 4 days at 20 °C).

##### Physiological Disorders and Sensory Quality

Flesh browning and flesh bleeding indices indicating chilling injury in coated and uncoated ‘Angeleno’ plums stored for 3 and 6 weeks at 1 °C followed by the 4-day shelf-life period at 20 °C are presented in Table 4. At harvest and after the first 3 weeks, plums showed no visible symptoms of physiological disorders. At the end of the experiment, a very low level of flesh browning was observed (index of 1.27 on a scale from 1 = none to 5 = severe) in uncoated plums, while this disorder was absent in coated plums (index around 1.00). However, these differences were not statistically significant (*p* > 0.05). Similar results were observed for flesh bleeding and although bleeding index was higher in uncoated fruit (index of 1.50 on a scale from 1 = none to 3 = severe) than in coated fruit (indexes from 1.00 to 1.24), these differences were not statistically significant.

The impact of antifungal CECs on sensory properties of cold-stored ‘Angeleno’ plums is shown in Table 4. Overall, the evaluated CECs maintained the sensory quality of plums throughout storage, with no significant differences compared to the control samples. At harvest and during storage, both coated and uncoated plums were evaluated as having a good external visual quality (values of 2.70–2.90 on a qualitative scale from 1 = bad to 3 = good). The overall flavor decreased from a score of 7.5 (good quality) at harvest to 6.1 (acceptable) at the end of the storage period, whereas off-flavor scores remained around 1.0 (absence) throughout the entire period. Regarding sensory or eating firmness, scores slightly decreased from 3.6 (firm) at harvest to 3.1 at the end of storage (moderately firm). Advantageously, significant differences in plum gloss (*p* < 0.05) were observed between coated and uncoated fruit after 3 and 6 weeks at 1 °C plus the shelf-life period at 20 °C (Table 5). Panelists found that all CEC-coated plums were glossier than uncoated plums, regardless of the coating formulation.

## 4. Discussion

There is currently a consensus on the need for more sustainable and safer methods for controlling postharvest BR of stone fruits that can reduce or eliminate the use of polluting synthetic chemical fungicides. The use of EOs and other extracts obtained from natural and renewable sources represents an eco-friendly and sustainable alternative for postharvest disease management [26]. The present research explored the antifungal activity of different pure volatiles, natural extracts, and EOs against *M. fructicola*, the most critical postharvest fungal pathogen affecting fresh plums. Moreover, their suitability as ingredients of HPMC-GMS-based CECs for BR control and postharvest quality preservation of plums were evaluated. To our knowledge, this is the first published study integrating in vitro assays to select natural antifungals effective against *M. fructicola*, in vivo assays with artificially inoculated plums using CECs as carriers for these natural agents, and quality assessments of coated fruit under simulated commercial conditions.

Thyme (*Thymus vulgaris*), oregano (*Origanum vulgare*), and SA (*S. montana*) EOs and their main components, such as carvacrol and thymol, were reported by several authors as highly effective antifungal agents against *M. fructicola* in in vitro assays [11,24,27,28,29,30]. However, as far as we are concerned, no reports are available about the in vitro antifungal activity of other EOs such as MY (*C. myrrha*) and LE (*C. citratus*) against *M. fructicola*, and only one report was found for CI (*C. zeylanicum*) applied as a volatile. Recently, Alvarez-García et al. [27] reported the inhibitory activity of several EOs in the vapor phase against three pathogenic species of *Monilinia* isolated from infected nectarines. These authors found that CI applied at 90.9 µL/L in Petri dishes allowed a complete growth inhibition of *M. fructicola* after a 5-day incubation period, which is in line with our results when expressed as volume of EO/volume of air in the headspace for comparison. It is well known that the biological activity of EOs is linked to their composition, which can vary depending on the plant source and extraction method [27,30]. The antifungal activity of CI was attributed to the presence of cinnamaldehyde and EU, among other constituents [27,31]. Sellamuthu et al. [24], using a similar volatile exposure assay as that in our study, found that thyme EO applied at 5 µL/plate exerted a fungistatic effect on *M. fructicola* with 100% mycelial growth inhibition, whereas peppermint (*Mentha piperita*) and citronella (*Cymbopogon nardus*) EOs inhibited by 80 and 65% the fungal growth, respectively. In comparison, all the volatiles and pure compounds evaluated in our study (SA, LE, CI, EU, and GE) proved to be more effective, with 100% growth inhibition and a demonstrated fungicidal effect at the same dose (5 µL/plate). It is worth mentioning that citronella and LE EOs are natural compounds derived from different species within the same genus, *Cymbopogum*, which differ in their chemical composition and, consequently, in their biological properties. In a recent study testing EOs activity by direct contact in agar dilution tests [30], the EO from another SA species (*Satureja hortensis*, rich in carvacrol) inhibited the in vitro mycelial growth of *M. fructicola* isolates with a minimal inhibitory concentration (MIC) of 500 µL/L, while thyme EO from the species *Thymus zigis* (rich in thymol) and oregano EOs (rich in carvacrol) were more effective, with MICs ranging from 200 to 250 µL/L. Likewise, our results also align with those reported by Verdeguer et al. [32], where the same commercial SA EO, containing carvacrol (24%) as main component and applied at 300 µg/mL, showed a 100% in vitro growth inhibition of plant pathogenic species of the genera *Fusarium*, *Alternaria*, and *Botrytis*.

GE is a natural aliphatic monoterpene, with a functional alcohol group in its organic composition, that exhibits broad antimicrobial activity. It is a main component of many EOs from various plants such as palmarose (*Cymbopogon martinii*), geranium (*Pelargonium graveolens*), and citronella (*C. nardus*), among others [33]. EU is a natural phenolic compound that can be isolated from the EOs of some herbal plants such as clove (*Eugenia caryophyllata*), *Cinnamomum* spp., and tulsi (*Ocimum tenuiflorum*) [34]. In a study by Tsao and Zhou [28], the in vitro antifungal activity of 22 naturally monoterpenoid compounds against *M. fructicola* and *B. cinerea* was evaluated. Among the pure volatile compounds studied, GE and EU were reported to be good growth inhibitors of both fungi, which is consistent with our present results. Thus, these authors reported that the addition to the culture medium of GE and EU at 100 µg/mL inhibited the mycelial growth of *M. fructicola* by 85 and 90%, respectively. However, in volatile exposure tests, these compounds only showed a moderate inhibitory effect (32–46% growth inhibition after 48 h) when 250 µg were applied in the lid of Petri dishes. In our study, a fungicidal (lethal) effect against *M. fructicola* was observed when applying a considerably higher dose (5 µL, equivalent to 4450 µg). The in vitro antifungal capacity of GE against other important fungal pathogens, such as *Rhizopus stolonifer* (Ehrenb.) Vuill. and *Alternaria alternata* (Fr.) Keissl., has also been documented [35,36]. In the case of MY EO, in vitro tests showed a lower antifungal efficacy compared to the rest of the EOs, probably due to the lower volatility of their components. The commercial MY EO used in this work contained curzerene (40.7%) and lindestrene (25.6%) as major components (data provided by the supplier). The antifungal properties of MY EO were previously demonstrated by Perveen et al. [37], who observed reductions in spore germination and mycelial growth of several phytopathogenic fungi in vitro, finding MIC values of 1.25 mL/L for *A. alternata*, *Fusarium oxysporum* Schltdl. *Fusarium solani* (Mart.) L. Lombard & Crous, and *Cladosporium* sp., which are similar to the effective concentrations of MY EO found in our study for the inhibition of *M. fructicola*. VA is a natural phenolic aldehyde with proven antibacterial and antifungal properties. According to Yang et al. [38], complete mycelial growth inhibition of *B. cinerea* and *A. alternata* (isolated from cherry tomatoes) was achieved in in vitro assays with the addition of VA to the culture medium at 2 g/L. However, its effect against *M. fructicola* had not yet been reported, and in our study a greater sensitivity was observed compared to other fungal species, with 100% growth inhibition and fungicidal effect at 0.31 g/kg. In previous studies by our group, the same natural antifungal agents tested in the present work were evaluated in vitro against *Penicillium digitatum* (Pers.) Sacc. and *Geotrichum citri-aurantii* (Ferraris) E.E. Butler, both postharvest fungal pathogens causing predominant diseases on citrus fruits [22,23]. In these studies, volatiles such as SA, CN, EU, and GE effectively inhibited the growth of both citrus pathogens. However, doses of 20–40 µL/plate were required to achieve 90–100% growth inhibition. In contrast, *M. fructicola* showed greater sensitivity to these antifungals, requiring lower doses (5 µL/plate) for complete radial growth inhibition and a fungicidal effect. In the case of LE, its effectiveness against *P. digitatum* was lower than that of the rest of EOs [22], while in the present work it was as effective against *M. fructicola* as the rest of volatile compounds. A similar trend was observed when testing GT, VA, and MY by the agar dilution method against the mentioned citrus pathogens [22,23], which, according to the present results, resulted in being more resistant to these antifungals than *M. fructicola*. The antifungal power of EOs is generally attributed to synergistic interactions between their chemical components, which are largely influenced by their hydrophobicity and ability to partition into microbial cell membranes [3]. Thus, these chemical agents can compromise the structure and functionality of fungal cell membranes, cell walls, and membrane proteins, leading to multiple detrimental effects such as increased membrane fluidity and permeability, interference with ion transport processes, leakage of intracellular components, inactivation of vital enzymes, and spore disruption, among others [27,39].

Based on the results obtained from in vitro tests and preliminary in vivo applications of various coating formulations, CI, LE, GE, and MY were selected as the most effective antifungal agents for further evaluation as CEC ingredients. For this, ‘Black Gold’ plums inoculated with *M. fructicola* 24 h before were coated with CECs enriched with the selected antifungal compounds at different concentrations and incubated at 20 °C to evaluate their curative activity. This is necessary because postharvest brown rot of stone fruits typically develops from latent infections produced in the field and, hence, commercial postharvest antifungal treatments must be able to control existing infections. The temperature chosen for this in vivo assay is commonly used to simulate fresh fruit shelf life and favors the growth of the pathogen, so results can be obtained faster than in cold storage experiments. On the other hand, the addition of natural extracts or EOs to coating-forming matrices could offer significant advantages over direct application of such substances by spraying or dipping onto fruit surface. Thus, coating application would favor a slower diffusion of the antifungal agents, ensuring effective concentrations on the fruit peel, a lower risk of phytotoxicity, and a lower impact on the fruit sensory properties [12]. As expected, the application of the CEC-based coating (without antifungal agents) to inoculated plums did not reduce BR throughout the incubation period at 20 °C (Figure 1), highlighting the need to functionalize the coatings with the addition of active antifungal substances to the emulsion formulation. In this trial, the effectiveness of EOs incorporated into the CECs was concentration-dependent and did not directly correlate with their inhibitory activity in the previous in vitro tests. For example, CI, which was as effective as GE and LE in impeding the in vitro mycelial growth of *M. fructicola*, failed to reduce BR development on plums when applied as a CEC ingredient. Such differences in the efficacy of EOs in in vitro and in vivo experiments have been previously reported [22,23] and can be mainly attributed to differences in the release rate of the active substance from the coating matrix to the fruit surface, compared to direct exposure in the culture medium. In this sense, numerous works in the literature describe the potential of biopolymers to encapsulate EOs and modulate their release in stand-alone films and edible coatings [40,41,42]. Among other factors, the release rate depends on the interaction between the EO and the polymer matrix. Thus, for example, Lian et al. [40] reported that the incorporation of xanthan gum, pullulan, gum tragacanth, or Arabic gum into chitosan-based composite films changed the release rate of thyme EO, being Arabic gum the most effective polymer to stabilize the EO in the film. This implied that films with higher release rates, even though effective against *M. fructicola* in in vitro studies, were not effective in controlling brown rot in in vivo trials with nectarines. This was attributed to the stronger chemical interactions between chitosan and Arabic gum compared to that of chitosan and the other polysaccharides. Also, the volatilization effects of the active substances or degradation due to interactions with other components of the coating matrix could affect the effectiveness of this type of antifungals by decreasing their effective concentration on the fruit surface. On the other hand, LE and GE effectively reduced BR incidence and severity during the 8-day incubation period when used at the highest concentration (4 g/kg), while the lowest concentration used for MY (5 g/kg) was the most effective. Overall, for each pathosystem, the ability of antifungal compounds in CECs to suppress or control disease development is influenced by multifaceted interactions between the host organism, the pathogen, and the environment (including the composite coating materials) that occur during the progression of the infection. Examples of effects of such interactions have been discussed in previous studies by our research group [3,14,22,23]. In the cold storage trial simulating commercial fruit handling, the effectiveness of the antifungal CECs decreased as storage time increased, resulting in lower reductions in disease incidence and severity compared to the uncoated control. These findings confirmed that the effect of these EOs, once integrated into the composite coating matrix, is rather fungistatic than fungicidal. Similar observations have been reported in prior research works with pectin-based CECs enriched with CI, EU, GE, or MY to control green mold caused by *P. digitatum* on cold-stored ‘Valencia’ oranges [22] and HPMC or gum Arabic-based coatings formulated with GE to control BR caused by *M. fructicola* on ‘Angeleno’ plums [14]. In these works, CECs containing GE significantly controlled disease on oranges or plums during commercial cold storage, but their effectiveness decreased with storage time and when fruits were transferred to 20 °C for shelf-life simulation, disease incidence and severity further increased, thus confirming the fungistatic action of the antifungal CECs.

Overall, our findings demonstrated the ability of HPMC-GMS-based emulsions containing GE (2 g/kg) or MY (5 g/kg) to reduce BR development on plums, both at room temperature and during long-term cold storage. Moreover, the coating CEC-LE significantly reduced BR severity, although it did not affect BR incidence. To our knowledge, the efficacy of biopolymer-based CECs enriched with volatile substances as antifungals for the control of BR caused by *M. fructicola* on plums has only been previously documented in a recent study by our group. In that research [14], CECs formulated with HPMC or gum Arabic combined with beeswax as lipidic component and supplemented with GE effectively reduced BR on artificially inoculated ‘Angeleno’ plums. On the other hand, the potential of CECs functionalized by the addition of MY or LE EOs to control BR and preserve plum quality is reported here for the first time. In the literature, scarce studies focused on improving the microbiological quality of Japanese plums after harvest by using antimicrobial CECs incorporating volatiles. Among them, a carnauba wax-based nanoemulsion containing LE EO (up to 30 g/kg) was reported to reduce the bacteria *Salmonella typhimurium* and *Escherichia coli* O157:H7 by up to 2.7 log units on pre-inoculated plums stored at either 4 or 25 °C [18]. Also, Choi et al. [19] reported that a HPMC-based coating formulated with bergamot and oregano EOs (20 g/kg) effectively reduced the total microbial counts and preserved the quality of ‘Formosa’ plums stored at either 5 or 23 °C. Andrade et al. [17] showed that gum Arabic coatings enriched with oregano and rosemary EOs delayed the onset and reduced the incidence of Rhizopus soft rot on plums during storage at both room (25 °C) and low (12 °C) temperatures. Moreover, another recent work by Jenneker et al. [21] reported the effect of gum Arabic-based coatings formulated with thymol or salicylic acid to control decay caused by *M. laxa* and *B. cinerea* on artificially inoculated ‘Angeleno’ plums during incubation at 23 °C. It was found that the CEC containing thymol did not significantly reduce disease incidence, although it effectively reduced disease severity.

In the present study, the effect of the antifungal CECs on the physicochemical properties and sensory attributes of ‘Angeleno’ plums was assessed after extended cold storage at 1 °C and a subsequent shelf-life period at 20 °C. Plum moisture loss during cold storage and commercialization can lead to significant economic losses and compromise fruit quality and external appearance. A protective effect of the HPMC-GMS formulation without antifungals (CEC treatment) against moisture loss was observed during the entire storage period, demonstrating its effectiveness as a water vapor barrier. CECs formulated with antifungal EOs also protected ‘Angeleno’ plums against weight loss after 3 weeks of cold storage, with an effect similar to that of the CEC coating. However, after 6 weeks plus shelf life, only the CEC and CEC-GE formulations maintained this effect. The incorporation of lipophilic components, GMS and EOs in this case, into the biopolymer matrix was expected to contribute to increasing the hydrophobicity of the coatings and, consequently, improve their water vapor barrier properties. In this sense, Choi et al. [19] reported that bergamot and oregano EOs enhanced the water barrier property of HPMC-based coatings and showed lower weight loss values of ‘Formosa’ plums stored at 5 and 23 °C, compared to HPMC without the EOs. However, it is necessary to highlight that specific interactions between polymer chains, lipid components, minor ingredients such as plasticizers and emulsifiers, and active compounds can promote changes in water permeability, which varies between CEC formulations with different composition [14,22]. In addition, other factors, such as cultivar, physiological state, and storage conditions, may influence the effectiveness of CECs in preserving weight loss and general fruit quality after harvest. For example, in a recent study, HPMC formulated with beeswax as a lipid component showed no effect on the weight loss of ‘Angeleno’ plums stored for 8 weeks at 1 °C followed by 3 days at 7 °C and 7 days at 20 °C, simulating transport and shelf-life conditions [14]. In contrast, a study by Gunaydin et al. [25] found that HPMC-beeswax coatings, with or without antifungal additives, were able to reduce the weight loss of ‘Friar’ plums after 22 days of cold storage at 1 °C plus a 5-day shelf-life period at 20 °C, with the most effective CECs containing paraben salts.

Plum postharvest softening is an important quality defect that reduces fruit shelf life, interferes with an appropriate commercialization, and directly affects consumer perception. Loss of cell wall integrity causes softening in fresh fruits, which may result from water loss and ripening [20]. During ripening, cell wall polysaccharides are hydrolyzed by enzymes such as pectin methylesterase and polygalacturonase resulting in loss of firmness [25]. In our study, uncoated plums showed the lowest firmness values, while virtually all CECs were able to reduce firmness loss after extended cold storage and shelf life. Nevertheless, the observed reductions in weight loss due to CEC application were not always correlated with lower firmness loss. Therefore, a combined effect of reduced water loss and delayed ripening, as a consequence of reduced respiration, may explain the observed firmness improvement on coated plums with respect control fruit. Several studies reported similar findings regarding firmness maintenance on coated fruit, which were explained by the ability of CECs to modify the internal atmosphere of the fruit with the consequent reduction in the activity of cell wall-degrading enzymes [43,44].

External color and juice SSC, TA, and MI (SSC/TA) are key indicators of stone fruit quality, determining flavor and greatly influencing the consumers’ purchasing decisions. Although some significant differences were observed between color parameters of uncoated and coated plums, CEC application did not adversely affect the natural color and aspect of ‘Angeleno’ plums. Likewise, plum internal quality was not influenced by the application of CECs throughout the entire storage period. TA losses in coated fruit would be expected to be delayed due to a reduction in respiration and consequent organic acid consumption [45]. In this work, slightly lower TA values were found in CEC-GE and CEC-MY coated plums compared to the control only after 3 weeks of cold storage plus shelf life, while no significant differences were observed between treatments at the end of storage. Coating composition and physiological aspects of the fruit are key factors affecting the fruit postharvest metabolic processes. Thus, some reports have shown that internal quality parameters such as SSC and TA in climacteric fruit are less dependent on ethylene than other ripening parameters such as firmness and color [46,47].

Typically, CECs act as a barrier to gas exchange on the surface of coated fruit, reducing internal O_2_ and increasing internal CO_2_ concentrations. This modification of the internal atmosphere can result in increased concentrations of fermentation volatiles such as ethanol and acetaldehyde [25]. These changes are greatly influenced by the composition and properties of the coatings, such as gas permeability, as well as the type of fruit and storage conditions. In our study, modifications in plum internal atmosphere caused by CEC application, particularly CEC-LE and CEC-GE, were evident, with considerably increased concentrations of ethanol and, to a lesser extent, of acetaldehyde. Similar findings were reported in previous studies with ‘Angeleno’ and ‘Friar’ plums coated with HPMC-based emulsions and subjected to long-term cold storage and shelf life [25,43].

Chilling injury in stone fruits generally manifests as flesh damage, including internal browning, flesh bleeding, translucency, gel breakdown, and mealiness. The appearance of these symptoms in cold-stored fruits is affected by several factors such as fruit cultivar, physiological stage, and storage conditions [2]. Among different cultivars, ‘Friar’, ‘Showtime’, and ‘Howard Sun’ plums are highly prone to developing chilling injury symptoms when fruit are transferred to 20 °C after cold storage at the optimum storage conditions of 0–1 °C [45]. In less susceptible cultivars such as ‘Angeleno’, the incidence and severity of chilling injury depends on the length of storage at less suitable temperatures (2–7 °C), which unfortunately are often reached during commercial transportation. In the present study, no evidence of chilling injury was found in either coated or uncoated plums, which could be attributed to the storage of the plums at the optimum temperature (1 °C). However, it is worth noting that other studies have reported the beneficial effect of CECs in reducing chilling injury in trials simulating a transport period at 5–7 °C (non-optimal temperature) after cold storage at 1 °C and prior to storage at 20 °C, simulating commercialization [14,43].

Sensory evaluation showed that the application of antifungal CECs formulated with HPMC-GMS and EOs had no adverse effects on any of the parameters evaluated. Thus, the fermentation volatile levels detected in coated samples were not high enough to be perceived as off-flavors by the sensory panel. Furthermore, the visual quality of the plums was considered good during storage, and fruit gloss was significantly superior on coated fruit, which can improve the consumer perception of fruit quality and positively influence the purchase decisions.

## 5. Conclusions

The present study demonstrates the suitability of HPMC-GMS-based ECs containing selected antifungal EOs as a safe and eco-friendly alternative to commercial waxes containing synthetic fungicides to control BR caused by *M. fructicola* and maintain postharvest quality of fresh plums. Among the different antifungal CECs tested, those formulated with GE or MY were the most effective as curative treatments against BR on artificially inoculated plums either incubated at room temperature or stored at 1 °C. All coated plums maintained higher firmness than uncoated control fruit, while CECs enriched with GE reduced fruit weight loss after 6 weeks of cold storage followed by 4 days of shelf life. In addition, the physicochemical and sensory quality of cold-stored ‘Angeleno’ plums was not adversely affected by CEC application, and increased gloss was observed in the peel of all coated fruits. Overall, the best results were obtained with the HPMC-GMS CEC formulated with 2 g/kg GE, which could be regarded as a promising alternative for reducing decay and extending the postharvest life of cold-stored plums. Nevertheless, further research might be devoted to improving the controlled release of volatile bioactive antifungals from the CEC matrix, optimizing the coating composition to further improve fruit weight loss reduction during cold storage, or exploring new biopolymers and lipids that may enhance the compatibility with the antifungal agents for better in vivo performance. In addition, the effect of these antifungal CECs could be tested with other plum cultivars or even other stone fruit species in order to broaden their spectrum of action and facilitate the commercial adoption of this type of CEC formulations by the industry.

## Figures and Tables

**Table 1:** Percentage inhibition of the mycelial radial growth of *Monilinia fructicola* caused by pure volatiles, essential oils, and natural extracts in in vitro assays with potato dextrose agar (PDA) plates incubated for 7 days at 25 °C.

Volatile Exposure Method				Agar Dilution Method			
Agent ^1^	Dose (µL)	Mycelial Growth Inhibition (%) ^2^		Agent ^1^	Dose (g/kg)	Mycelial Growth Inhibition (%) ^2^	
EU	5	100.0 ± 0.0	a # ^3^	GT	5.00	0.0 ± 0.0	f
	10	100.0 ± 0.0	a #		10.00	47.0 ± 0.4	e
GE	5	100.0 ± 0.0	a #		20.00	66.0 ± 0.5	d
	10	100.0 ± 0.0	a #	VA ^4^	0.31	100.0 ± 0.0	a # ^3^
CI	5	100.0 ± 0.0	a #		0.62	100.0 ± 0.0	a #
	10	100.0 ± 0.0	a #		1.25	100.0 ± 0.0	a #
LE	5	100.0 ± 0.0	a #	MY ^4^	0.62	93.8 ± 1.2	c
	10	100.0 ± 0.0	a #		1.25	95.7 ± 0.7	b
SA	5	100.0 ± 0.0	a #		2.50	99.0 ± 0.6	a #
	10	100.0 ± 0.0	a #				

^1^ EU: eugenol; GE: geraniol; SA: savory essential oil (EO); CI: cinnamon EO; LE: lemongrass EO; GT: green tea extract; VA: vanillin; MY: myrrh EO. ^2^ Data are mean value ± standard error (SE) (n = 5). Different letters in the same column indicate significant differences between treatments according to Fisher’s Protected LSD test (*p* < 0.05) applied after an ANOVA. ^3^ # indicates fungicidal effect. ^4^ For these antifungal agents, percentage inhibition calculated with respect to control plates containing PDA amended with DMSO at 2.5 g/kg.

**Table 2:** Flesh firmness and peel color of ‘Angeleno’ plums treated with antifungal composite edible coatings and stored at 1 °C for 3 and 6 weeks followed by a 4-day shelf-life period at 20 °C.

Physical Properties		Storage Conditions	Treatment ^1^				
			Control	CEC	CEC-LE (4 g/kg)	CEC-GE (2 g/kg)	CEC-MY (5 g/kg)
Firmness (N) ^2^		At harvest	17.13 ± 1.10				
		3 weeks 1 °C + 4 days 20 °C	16.44 ± 0.81 ^a^	16.93 ± 0.97 ^a^	15.43 ± 1.05 ^a^	14.63 ± 0.89 ^a^	14.56 ± 1.01 ^a^
		6 weeks 1 °C + 4 days 20 °C	12.00 ± 0.93 ^c^	14.72 ± 0.64 ^ab^	15.66 ± 0.66 ^a^	13.21 ± 0.55 ^bc^	16.76 ± 1.00 ^a^
Peel Color ^2^	L*	At harvest	30.79 ± 0.70				
		3 weeks 1 °C + 4 days 20 °C	28.94 ± 0.45 ^a^	25.17 ± 0.27 ^b^	25.01 ± 0.35 ^b^	25.78 ± 0.46 ^b^	25.20 ± 0.41 ^b^
		6 weeks 1 °C + 4 days 20 °C	28.28 ± 0.37 ^a^	25.01 ± 0.28 ^b^	24.99 ± 0.23 ^b^	24.62 ± 0.12 ^b^	24.78 ± 0.26 ^b^
	a*	At harvest	6.37 ± 0.35				
		3 weeks 1 °C + 4 days 20 °C	10.79 ± 1.24	9.63 ± 0.99	9.55 ± 1.31	10.43 ± 1.36	8.10 ± 0.82 ^ns^
		6 weeks 1 °C + 4 days 20 °C	5.46 ± 0.83 ^b^	8.61 ± 0.76 ^a^	8.22 ± 0.90 ^a^	7.04 ± 0.49 ^ab^	7.82 ± 0.82 ^a^
	b*	At harvest	−2.82 ± 0.31				
		3 weeks 1 °C + 4 days 20 °C	0.13 ± 0.70 ^b^	1.38 ± 0.42 ^ab^	2.08 ± 0.65 ^a^	2.10 ± 0.75 ^a^	1.01 ± 0.34 ^ab^
		6 weeks 1 °C + 4 days 20 °C	−2.56 ± 0.40 ^b^	0.81 ± 0.24 ^a^	1.20 ± 0.34 ^a^	0.67 ± 0.16 ^a^	0.95 ± 0.29 ^a^
	C*	At harvest	7.15 ± 0.28				
		3 weeks 1 °C + 4 days 20 °C	11.37 ± 1.17	9.81 ± 1.04	9.87 ± 1.43	10.81 ± 1.49	8.22 ± 0.86 ^ns^
		6 weeks 1 °C + 4 days 20 °C	6.54 ± 0.72	8.69 ± 0.78	8.35 ± 0.95	7.10 ± 0.49	7.91 ± 0.85 ^ns^
	*h*°	At harvest	335.36 ± 3.11				
		3 weeks 1 °C + 4 days 20 °C	351.38 ± 5.29 ^b^	5.15 ± 1.71 ^a^	7.93 ± 1.70 ^a^	5.45 ± 2.69 ^a^	4.89 ± 1.52 ^a^
		6 weeks 1 °C + 4 days 20 °C	327.45 ± 4.59 ^b^	3.69 ± 1.26 ^a^	5.67 ± 1.37 ^a^	4.66 ± 1.43 ^a^	4.57 ± 1.24 ^a^

^1^ Control: uncoated fruit. CEC: composite edible coating; LE: lemongrass essential oil (EO); GE: geraniol; MY: myrrh EO. ^2^ Data are mean value ± standard error (SE) (n = 20). For each quality parameter and storage period, different letters and ‘ns’ in each row indicate significant and non-significant differences among treatments, respectively, according to Fisher’s Protected LSD test (*p* < 0.05) applied after an ANOVA.

**Table 3:** Internal quality and volatile content of ‘Angeleno’ plums treated with antifungal composite edible coatings and stored at 1 °C for 3 and 6 weeks followed by a 4-day shelf-life period at 20 °C.

Juice Quality ^2^	Storage Conditions	Treatment ^1^				
		Control	CEC	CEC-LE (4 g/kg)	CEC-GE (2 g/kg)	CEC-MY (5 g/kg)
Titratable Acidity (TA; g/L malic acid)	At harvest	8.62 ± 0.26				
	3 weeks 1 °C + 4 days 20 °C	8,37 ± 0.17 ^a^	8.49 ± 0.20 ^a^	8.08 ± 0.04 ^a^	7.45 ± 0.13 ^b^	7.25 ± 0.20 ^b^
	6 weeks 1 °C + 4 days 20 °C	7.21 ± 0.07	6.54 ± 0.23	6.91 ± 0.16	6.43 ± 0.24	7.04 ± 0.15 ^ns^
Soluble Solids Content (SSC; °Brix)	At harvest	14.67 ± 0.23				
	3 weeks 1 °C + 4 days 20 °C	14.33 ± 0.24	14.20 ± 0.05	14.32 ± 0.22	14.05 ± 0.21	14.75 ± 0.28 ^ns^
	6 weeks 1 °C + 4 days 20 °C	14.23 ± 0.65	12.82 ± 0.02	13.25 ± 0.28	12.73 ± 0.16	14.18 ± 0.79 ^ns^
Maturity Index (MI = SSC/TA)	At harvest	17.07 ± 0.76				
	3 weeks 1 °C + 4 days 20 °C	17.13 ± 0.10 ^c^	16.75 ± 0.44 ^c^	17.71 ± 0.23 ^bc^	18.87 ± 0.41 ^b^	20.36 ± 0.60 ^a^
	6 weeks 1 °C + 4 days 20 °C	20.56 ± 0.35	19.66 ± 0.71	19.18 ± 0.18	19.83 ± 0.56	20.13 ± 0.83 ^ns^
**Volatile content ^2^**						
Ethanol (mg/L)	At harvest	ND				
	3 weeks 1 °C + 4 days 20 °C	8.6 ± 3.8 ^c^	7.4 ± 2.6 ^c^	34.3 ± 2.6 ^a^	8.5 ± 0.6 ^c^	14.3 ± 2.0 ^b^
	6 weeks 1 °C + 4 days 20 °C	6.5 ± 2.1 ^c^	37.4 ± 10.3 ^c^	311.9 ± 53.6 ^a^	242.7 ± 34.6 ^a^	130.5 ± 11.0 ^b^
Acetaldehyde (mg/L)	At harvest	1.0 ± 0.1				
	3 weeks 1 °C + 4 days 20 °C	2.6 ± 0.5	1.5 ± 0.2	2.2 ± 0.5	2.4 ± 0.2	1.9 ± 0.2 ^ns^
	6 weeks 1 °C + 4 days 20 °C	2.3 ± 0.8 ^c^	4.4 ± 0.8 ^bc^	7.7 ± 1.1 ^a^	4.8 ± 0.5 ^b^	4.2 ± 0.4 ^bc^

^1^ Control: uncoated fruit. CEC: composite edible coating; LE: lemongrass essential oil (EO); GE: geraniol; MY: myrrh EO. ^2^ Data are mean value ± standard error (SE) (n = 3). For each quality parameter and storage period, different letters and ‘ns’ in each row indicate significant and non-significant differences among treatments, respectively, according to Fisher’s Protected LSD test (*p* < 0.05) applied after an ANOVA. ND: not determined.

**Table 4:** Physiological disorders and sensorial quality of ‘Angeleno’ plums treated with antifungal edible coatings and stored at 1 °C for 3 and 6 weeks followed by a 4-day shelf-life period at 20 °C.

Physiological Disorders ^2,3^	Storage Conditions	Treatment ^1^				
		Control	CEC	CEC-LE (4 g/kg)	CEC-GE (2 g/kg)	CEC-MY (5 g/kg)
Flesh Browning Index	At harvest	1.00 ± 0.00				
	3 weeks 1 °C + 4 days 20 °C	1.00 ± 0.00	1.00 ± 0.00	1.00 ± 0.00	1.00 ± 0.00	1.00 ± 0.00 ns
	6 weeks 1 °C + 4 days 20 °C	1.27 ± 0.12	1.07 ± 0.03	1.00 ± 0.00	1.03 ± 0.03	1.10 ± 0.10 ns
Flesh Bleeding Index	At harvest	1.00 ± 0.00				
	3 weeks 1 °C + 4 days 20 °C	1.07 ± 0.03	1.00 ± 0.00	1.00 ± 0.00	1.00 ± 0.00	1.00 ± 0.00 ns
	6 weeks 1 °C + 4 days 20 °C	1.50 ± 0.12	1.17 ± 0.04	1.00 ± 0.00	1.17 ± 0.08	1.24 ± 0.12 ns
**Sensory attributes ^2,4^**						
Visual Quality (whole fruit)	At harvest	2.88 ± 0.13				
	3 weeks 1 °C + 4 days 20 °C	2.70 ± 0.15	2.70 ± 0.15	2.70 ± 0.15	2.90 ± 0.10	2.70 ± 0.21 ns
	6 weeks 1 °C + 4 days 20 °C	2.80 ± 0.11	2.78 ± 0.15	2.89 ± 0.11	2.89 ± 0.11	2.89 ± 0.11 ns
Overall Flavor	At harvest	7.50 ± 0.38				
	3 weeks 1 °C + 4 days 20 °C	6.78 ± 0.33	6.30 ± 0.33	6.67 ± 0.37	6.56 ± 0.37	6.40 ± 0.37 ns
	6 weeks 1 °C + 4 days 20 °C	6.11 ± 0.35	6.11 ± 0.45	6.33 ± 0.33	6.33 ± 0.29	6.22 ± 0.22 ns
Off-Flavor	At harvest	1.00 ± 0.00				
	3 weeks 1 °C + 4 days 20 °C	1.20 ± 0.13	1.10 ± 0.10	1.10 ± 0.10	1.20 ± 0.13	1.40 ± 0.22 ns
	6 weeks 1 °C + 4 days 20 °C	1.11 ± 0.11	1.22 ± 0.22	1.11 ± 0.11	1.11 ± 0.11	1.00 ± 0.00 ns
Eating Firmness	At harvest	3.63 ± 0.18				
	3 weeks 1 °C + 4 days 20 °C	3.40 ± 0.16	4.10 ± 0.18	3.50 ± 0.17	3.90 ± 0.23	3.30 ± 0.26 ns
	6 weeks 1 °C + 4 days 20 °C	3.11 ± 0.31	3.33 ± 0.24	3.00 ± 0.29	3.22 ± 0.22	3.67 ± 0.17 ns

^1^ Control: uncoated fruit. CEC: composite edible coating; LE: lemongrass essential oil (EO); GE: geraniol; MY: myrrh EO. ^2^ Data are mean value ± standard error (SE), n = 3 for physiological disorder scores, n = 10 for sensory scores. For each quality parameter and storage period, ‘ns’ in each row indicate non-significant differences among treatments according to Fisher’s Protected LSD test (*p* < 0.05) applied after an ANOVA. ^3^ Browning index rated from 1 (absence) to 5 (extreme severity); bleeding index from 1 (absence) to 3 (severe). ^4^ Visual quality (external appearance) was scored with 1 (bad), 2 (acceptable) or 3 (good). Overall flavor rated from 1 (very poor) to 9 (optimal); off-flavors from 1 (absence) to 5 (very pronounced); Eating (sensory) firmness from 1 (very soft) to 5 (very firm).

**Table 5:** Ranked gloss of ‘Angeleno’ plums treated with antifungal edible coatings and stored at 1 °C for 3 and 6 weeks followed by a 4-day shelf-life period at 20 °C.

Gloss Rank ^1^	Storage Conditions			
	3 Weeks 1 °C + 4 Days 20 °C		6 Weeks 1 °C + 4 Days 20 °C	
Glossiest Fruit	CEC-LE (4 g/kg)	a	CEC-GE (2 g/kg)	a
	CEC	a	CEC-LE (4 g/kg)	a
	CEC-GE (2 g/kg)	a	CEC-MY (5 g/kg)	a
	CEC-MY (5 g/kg)	a	CEC	a
Least Glossy Fruit	Control	b	Control	b

^1^ Control: uncoated fruit. CEC: composite edible coating; LE: lemongrass essential oil (EO); GE: geraniol; MY: myrrh EO. Treatments in columns with different letters are significantly different according to Friedman test (*p* < 0.05) (n = 10).

## Data Availability

The original contributions presented in the study are included in the article. Further inquiries can be directed to the corresponding author.

## References

[1] Wan X., Lin X., Zhang Y., Luo D., Peng J., Huang H., Ding X., Dong X. (2024). Revealing the crucial role of cuticular wax components in postharvest chilling injury of plums (*Prunus salicina* Lindl.). Sci. Hortic..

[2] Crisosto C.H., Mitchell F.G., Ju Z. (1999). Susceptibility to chilling injury of peach, nectarine, and plum cultivars grown in California. HortScience.

[3] Usall J., Casals C., Sisquella M., Palou L., De Cal A. (2015). Alternative technologies to control postharvest diseases of stone fruits. Stewart Postharv. Rev..

[4] Martini C., Mari M., Bautista-Baños S. (2014). *Monilinia fructicola*, *Monilinia laxa* (Monilinia Rot, Brown Rot). Postharvest Decay: Control Strategies.

[5] Lopez-Reyes J.G., Spadaro D., Prelle A., Garibaldi A., Gullino M.L. (2013). Efficacy of plant essential oils on postharvest control of rots caused by fungi on different stone fruits in vivo. J. Food Prot..

[6] Hassani A., Fathi Z., Ghosta Y., Abdollahi A., Meshkatalsadat M.H., Marandi R.J. (2012). Evaluation of plant essential oils for control of postharvest brown and gray mold rots on apricot. J. Food Saf..

[7] Min D., Wu H., Xu M., Leng P., Sun J., Liu Y.-G. (2025). Antifungal and mechanism of rose essential oil against *Monilinia fructicola* caused brown rot of peach fruit. Postharvest Biol. Technol..

[8] El Khetabi A., Lahlali R., Askarne L., Ezrari S., El Ghadaroui L., Tahiri A., Hrustić J., Amiri S. (2020). Efficacy assessment of pomegranate peel aqueous extract for brown rot (*Monilinia* spp.) disease control. Physiol. Mol. Plant Pathol..

[9] Lazar-Baker E.E., Hetherington S.D., Ku V.V., Newman S.M. (2011). Evaluation of commercial essential oil samples on the growth of postharvest pathogen *Monilinia fructicola* (G. Winter) Honey. Lett. Appl. Microbiol..

[10] Garello M., Schiavon G., Spadaro D. (2023). Efficacy of biofumigation with essential oils in the control of postharvest rots on nectarines. Acta Hortic..

[11] Santoro K., Maghenzani M., Chiabrando V., Bosio P., Gullino M.L., Spadaro D., Giacalone G. (2018). Thyme and savory essential oil vapor treatments control brown rot and improve the storage quality of peaches and nectarines, but could favor gray mold. Foods.

[12] Sánchez-Silva J.M., López-García U.M., Gutierrez-Martinez P., Flores-Ramírez A.Y., Ramos-Bell S., Moreno-Hernández C., Rivas-García T., González-Estrada R.R. (2025). Bionanocomposite coating film technologies for disease management in fruits and vegetables. Horticulturae.

[13] Karaca H., Pérez-Gago M.B., Taberner V., Palou L. (2014). Evaluating food additives as antifungal agents against *Monilinia fructicola* in vitro and in hydroxypropyl methylcellulose-lipid composite edible coatings for plums. Int. J. Food Microbiol..

[14] Asgarian Z.S., Palou L., Souza R.F.L.D., Quintanilla P.G., Taberner V., Karimi R., Pérez-Gago M.B. (2023). Hydroxypropyl methylcellulose and gum Arabic composite edible coatings amended with geraniol to control postharvest brown rot and maintain quality of cold-stored plums. Foods.

[15] More P.R., Pegu K., Arya S.S. (2022). Post-harvest application of micellar pomegranate peel extract (MPPE) enriched starch-casein composite coating to preserve the plum (*Prunus salicina* L.) fruit during cold and ambient storage. J. Food Process. Preserv..

[16] Martínez-Romero D., Zapata P.J., Guillén F., Paladines D., Castillo S., Valero D., Serrano M. (2017). The addition of rosehip oil to Aloe gels improves their properties as postharvest coatings for maintaining quality in plum. Food Chem..

[17] Andrade S.C.A., Baretto T.A., Arcanjo N.M.O., Madruga M.S., Meireles B., Cordeiro Â.M.T., Barbosa de Lima M.A., de Souza E.L., Magnani M. (2017). Control of Rhizopus soft rot and quality responses in plums (*Prunus domestica* L.) coated with gum arabic, oregano and rosemary essential oils. J. Food Process. Preserv..

[18] Kim I.H., Lee H., Kim J.E., Song K.B., Lee Y.S., Chung D.S., Min S.C. (2013). Plum coatings of lemongrass oil-incorporating carnauba wax-based nanoemulsion. J. Food Sci..

[19] Choi W.S., Singh S., Lee Y.S. (2016). Characterization of edible film containing essential oils in hydroxypropyl methylcellulose and its effect on quality attributes of ‘Formosa’ plum (*Prunus salicina* L.). LWT.

[20] Fawole O.A., Riva S.C., Opara U.L. (2020). Efficacy of edible coatings in alleviating shrivel and maintaining quality of Japanese plum (*Prunus salicina* Lindl.) during export and shelf life conditions. Agronomy.

[21] Jenneker N., Silué Y., Julia Meitz-Hopkins J.C., Lennox C.L., Opara U.L., Fawole O.A. (2024). Gum Arabic-incorporated thymol/salicylic acid composite coatings control grey mould and brown rot in ‘Angeleno’ plums. Eur. J. Plant Pathol..

[22] Alvarez M.V., Palou L., Taberner V., Fernández-Catalán A., Argente-Sanchis M., Pitta E., Pérez-Gago M.B. (2022). Natural pectin-based edible composite coatings with antifungal properties to control green mold and reduce losses of ‘Valencia’ oranges. Foods.

[23] Alvarez M.V., Pérez-Gago M.B., Taberner V., Settier-Ramírez L., Martínez-Blay V., Palou L. (2023). Postharvest application of novel bio-based antifungal composite edible coatings to reduce sour rot and quality losses of ‘Valencia’ oranges. Coatings.

[24] Sellamuthu P.S., Sivakumar D., Soundy P. (2013). Antifungal activity and chemical composition of thyme, peppermint and citronella oils in vapor phase against avocado and peach postharvest pathogens. J. Food Saf..

[25] Gunaydin S., Karaca H., Palou L., De La Fuente B., Pérez-Gago M.B. (2017). Effect of hydroxypropyl methylcellulose-beeswax composite edible coatings formulated with or without antifungal agents on physicochemical properties of plums during cold storage. J. Food Qual..

[26] Moradinezhad F., Ranjbar A. (2023). Advances in postharvest diseases management of fruits and vegetables: A review. Horticulturae.

[27] Álvarez-García S., Moumni M., Romanazzi G. (2023). Antifungal activity of volatile organic compounds from essential oils against the postharvest pathogens *Botrytis cinerea*, *Monilinia fructicola*, *Monilinia fructigena*, and *Monilinia laxa*. Front. Plant Sci..

[28] Tsao R., Zhou T. (2000). Antifungal activity of monoterpenoids against postharvest pathogens *Botrytis cinerea* and *Monilinia fructicola*. J. Essent. Oil Res..

[29] Elshafie H.S., Mancini E., Camele I., Martino L.D., De Feo V. (2015). In vivo antifungal activity of two essential oils from Mediterranean plants against postharvest brown rot disease of peach fruit. Ind. Crops Prod..

[30] Chang Y., Boukari W., Riley S.S., Harmon P.F., Sarkhosh A., Brecht J.K. (2024). In Vitro antifungal activity of white thyme, oregano, and savory oils against five *Monilinia fructicola* isolates from the Southeastern United States. Plant Health Prog..

[31] Combrinck S., Regnier T., Kamatou G.P.P. (2011). In vitro activity of eighteen essential oils and some major components against common postharvest fungal pathogens of fruit. Ind. Crops Prod..

[32] Verdeguer M., Roselló J., Castell V., Llorens J.A., Santamarina M.P. (2020). Cherry tomato and persimmon kaki conservation with a natural and biodegradable film. Curr. Res. Food Sci..

[33] de Lira M.H.P., de Andrade F.P., Moraes G.F.Q., Macena G.d.S., Pereira F.d.O., Lima I.O. (2020). Antimicrobial activity of geraniol: An integrative review. J. Essent. Oil Res..

[34] Khalil A.A., Rahman U.U., Khan M.R., Sahar A., Mehmood T., Khan M. (2017). Essential oil eugenol: Sources, extraction techniques and nutraceutical perspectives. RSC Adv..

[35] Zhou L., Zhang Z., Wei M., Xie Y., He S., Shi H., Lin Z. (2019). Evaluation of the antifungal activity of individual and combined monoterpenes against *Rhizopus stolonifer* and *Absidia coerulea*. Environ. Sci. Pollut. Res..

[36] Chutia M., Deka Bhuyan P., Pathak M.G., Sarma T.C., Boruah P. (2009). Antifungal activity and chemical composition of Citrus reticulata Blanco essential oil against phytopathogens from North East India. LWT-Food Sci. Technol..

[37] Perveen K., Bokhari N.A., Siddique I., Al-Rashid S.A.I. (2018). Antifungal activity of essential oil of *Commiphora molmol* oleo gum resin. J. Essent. Oil Bear. Plants.

[38] Yang J., Chen Y.Z., Wu Y.-X., Li T., Zhang Y.-D., Wang S.R., Zhang G.C., Zhang J. (2021). Inhibitory effects and mechanisms of vanillin on gray mold and black rot of cherry tomatoes. Pestic. Biochem. Physiol..

[39] Spadaro D., Banani H., Santoro K., Garibaldi A., Gullino M.L. (2021). Essential oils to control postharvest diseases of apples and peaches: Elucidation of the mechanism of action. Acta Hortic..

[40] Lian H., Shi J., Zhang X., Peng Y., Meng W., Pei L. (2022). Effects of different kinds of polysaccharides on the properties and inhibition of *Monilinia fructicola* of the thyme essential oil-chitosan based composite films. Food Sci. Technol..

[41] Heidary L., Nourbakhsh H., Javanmardi Z., Saba M.K. (2025). Biopolymer-enhanced nanoemulsions for controlled release of thyme: Impact on strawberry shelf life and quality. J. Agric. Food Res..

[42] Xiao R., Feng Y., Zhang X., Jiang Z., Chen W., Ding X., Yang G., Yang L. (2025). Using encapsulated essential oils to control food spoilage: Encapsulation techniques, release methods, and preservation strategies—A comprehensive review. Food Control.

[43] Navarro-Tarazaga M.L., Sothornvit R., Pérez-Gago M.B. (2008). Effect of plasticizer type and amount on hydroxypropyl methylcellulose- beeswax edible film properties and postharvest quality of coated plums (Cv. Angeleno). J. Agric. Food Chem..

[44] Valero D., Díaz-Mula H.M., Zapata P.J., Guillén F., Martínez-Romero D., Castillo S., Serrano M. (2013). Effects of alginate edible coating on preserving fruit quality in four plum cultivars during postharvest storage. Postharvest Biol. Technol..

[45] Riva S.C., Opara U.O., Fawole O.A. (2020). Recent developments on postharvest application of edible coatings on stone fruit: A review. Sci. Hortic..

[46] Johnston J.W., Gunaseelan K., Pidakala P., Wang M., Schaffer R.J. (2009). Co-ordination of early and late ripening events in apples is regulated through differential sensitivities to ethylene. J. Exp. Bot..

[47] Pech J.C., Bouzayen M., Latché A.J.P.S. (2008). Climacteric fruit ripening: Ethylene-dependent and independent regulation of ripening pathways in melon fruit. Plant Sci..

